# Dr. C. C. Allen's Letter in Reply to Our Remarks in Vol. 6, No. 2, upon the "Resolutions of the American Society of Dental Surgeons, in Relation to the Use of Amalgams for Filling Teeth

**Published:** 1846-06

**Authors:** John Lovejoy

**Affiliations:** New York


THE AMERICAN

### JOURNAL OF DENTAL SCIENCE.

Vol. VI.] JUNE, 1846. [No. 4.

### ARTICLE I.

### *Dr. C. C. Allen's Letter in Reply to our Remarks in Vol. 6, No. 2, upon the "Resolutions of the American Society of Dental Surgeons, in Relation to the Use of Amalgams for Filling Teeth.*

We have been long wishing to hear through the Journal, from some of those who take exceptions to the action of the Society, upon this subject, and we are certainly glad that as clever a man as is our correspondent, has volunteered to set forth the objections to the views and action of the Society, and to defend the position taken by those who dissent from these views and the corresponding action. Few men would have managed this subject more adroitly, or come nearer to making a good case out of a bad one.

Such is the ingenuity displayed, and so fascinating is his style, that its first reading well nigh forced us to conclude we had hitherto been in error upon this subject. But we were not quite willing hastily to relinquish views long entertained. We therefore gave it a second and more careful reading, and, although we lost none of our admiration for the style and temper in which it was written, yet we discovered much more from which we feel bound to dissent.

This subject has already occupied so much [illegible]ace in the Journal, that we feel somewhat reluctant either to ta[illegible]

or our subscribers with its farther discussion; yet as the forthcoming number immediately precedes the next annual meeting of the Society, at which we hope this subject will be finally disposed of, we shall embrace this opportunity to notice briefly some of the positions taken by Dr. Allen, in the letter above alluded to, in behalf of himself and friends.

His very first position, and one upon which his whole argument rests, is a position from which we beg leave most unqualifiedly to dissent. We cannot, indeed, refrain from expressing our unmixed surprise, that any one could take this position, after all that has been said and written upon this subject. He says, "First let me inquire what was the ground upon which the *American Society of Dental Surgeons* first took their action against amalgams?" The doctor answers his own question, by first telling us what was *not* the ground, and secondly, by informing us what it *was*. "It was *not*," he affirms, "that a majority believed that amalgams were, in all, or in a very large proportion of cases, in which they are carefully used, productive of bad results, either to the teeth or the constitution"* * * *. Secondly, he informs us in relation to the ground upon which the Society *did* take up arms against amalgams for plugging teeth. His language is as follows: "they," (the Society,) "had seen that there was a large class of men in the country, calling themselves dentists, who, either through ignorance or empiricism, were using and recommending amalgams, as better than gold, or tin, in cases where only gold was admissible, and that *these* men were doing great injury to their patients, and bringing reproach upon the dental science, and upon all practicing dentists, the honest and intelligent, as well as the ignoramus and the empiric. It was a measure of self-defence—they saw that, as a whole, taking the operations of the good and the bad together, the amalgam was doing infinitely more hurt in the hands of the latter, than good in those of the former, and like the reformers in the noble cause of temperance, they resolved that henceforward they would have nothing to do with it."

These very extraordinary assertions in relation to the doctrines held by the American Society, relative to the use of amal- [illegible] the time, and since their first action in regard to them, is to us, we confess, a piece of information. We had supposed that we had kept pace somewhat with the doings of this body, but if Dr. Allen informs us correctly, we are altogether in the back ground. Not only so, but if the doctrines of the Society are in fact as they are set forth by the gentleman above named, we shall not only agree with him in the opinion that "the Society have transcended their powers," in this prohibitory act, but they have overleaped the bounds of common sense and common prudence. But before coming to so rash a conclusion, let us examine the sentiments which have been from time to time expressed by the Society, and which, unfortunately for Dr. Allen's position, have been duly recorded.

The first specific action or expression of sentiments of the Society we find recorded upon this subject, are embodied in a resolution passed at their annual meeting, held in Philadelphia, Aug. 1841, and is as follows: "The committee, Drs. E. Parmly, E. Baker, S. Brown, C. A. Harris and J. Parmly, to whom the duty of reporting on the use of lithodeon, mineral paste, and all other substances of which mercury is an ingredient, for stopping teeth, reported, that the use of all such articles *are hurtful both to the teeth and every part of the mouth*, and that there was *no tooth* in which caries in it could be arrested, and the organ rendered serviceable by being filled, in which *gold* could not be employed. The report was *unanimously* adopted by the *Society.*" Now it strikes us that this resolution does not tally very well with the statements above quoted from Dr. Allen's letter. The above resolution most clearly and most *unqualifiedly* sets forth a two-fold objection to this article being *ever* employed by *any* one. First, the resolution declares the article "hurtful both to the teeth and every part of the mouth;" and secondly, that no exigency can occur, where its use is demanded, or "where gold could not be used."

Again, in Aug. 1843, the Society *unanimously* declare "the use of mineral paste, in plugging carious teeth, to be *malpractice.*" These very sentiments were again repeated, more strongly if possible, at the annual meeting in 1844 and in '45, and in every case these sentiments were adopted, *unanimously and without qualification.*

How Dr. Allen can, then, in the "face and eyes" of this record, declare that the Society did not take action against amalgams on the ground of believing them hurtful in all or in a very large proportion of cases, we cannot readily see.

But he informs us, secondly, that the Society *did* set their faces against it because it was used by many who did so through "ignorance or empiricism," and because it was doing more *harm* in the hands of the empiric, than *good* in the hands of the judicious practitioner. Although, after quoting the above resolutions of the Society, nothing more need be said to show that the reasons assigned by Dr. Allen, for the action of the Society against amalgams, are not the true ones, yet we will for a moment suppose them such, and examine some of the conclusions which must inevitably follow. We fear that Dr. Allen himself would shrink from many of the consequences of his own suppositions. For a Society to proscribe any article, simply because it does more mischief in the hands of quacks, than good in the hands of an opposite class, would be the height of absurdity, and proof positive that such a Society was incapable of bettering any cause, however good their intentions.

On such a supposition, well may Dr. Allen suppose that "*lobelia inflata*" would be unjustly condemned and proscribed, as a noxious weed. The fact is, that proscription based upon such grounds, would strike from the materia medica *every* powerful remedy which now has a place there. "Lobelia" would by no means occupy a front rank in the march to exile. It can hardly be doubted that even at the present time, when the science of dentistry has been brought, in many of its departments, well nigh to perfection, a time when dental surgery can boast of scattering blessings on every hand, that more misery is caused by *bad* operations, than there is benefit conferred by *good* ones. Dentistry might therefore, in one sense, be regarded as a positive evil, and on Dr. Allen's supposition, it would be just as reasonable for a Society to proscribe the *whole* practice as *malpractice*, as on his supposition to claim they had condemned a single item, on the self same principle.

We therefore assert, without fear of contradiction, that neither our correspondent, nor any one else, can find a single paragraph or sentence on record, which will for a moment countenance the supposition that the American Society of Dental Surgeons condemned and proscribed amalgams, on any other supposition than that their tendency is *uniformly* prejudicial both to the teeth and mouth—that they cannot, as has been clearly demonstrated, be efficient in arresting decay—that they are never called for by any exigency whatever, and that they are liable, under peculiar circumstances, (which no man can foresee,) to produce dangerous and even fatal results.* We say, then, that they have never, as a body, recognized any "*judicious use*" of amalgams.

Any other supposition is not only contrary to every record upon this subject, but is wholly *incompatible with their action in respect to it.* Nor have they recognized any class of cases, which its advocates denominate "*certain cases*," as constituting an exception to their sentiments in regard to amalgams. We know a man of some repute, who claims that *he* only uses amalgams in "certain cases." We have in our possession drawings of several mouths as left by him. One of them may be thus described: On the under jaw, this patient had lost the crowns of the first and second molars on the left side; the roots of each remained. In the anterior side of the third molar, and also the posterior side of the second bicuspid, were very large cavities. Not only were these cavities filled with amalgam, but also the intermediate space, to a level with the tops of the teeth on either side, making a perfect wall, bounded on the one end by the sapient, and on the other by the second bicuspid, and resting on the gum, and the end of the fangs of the first and second molars. The cement had remained in this situation some three or four weeks, when we were consulted in respect to the case. At this time, extensive ulceration had occurred in the adjacent soft parts, and a very copious discharge of offensive pus was constantly going on. But we have described this case sufficiently for our purpose. The operator who placed this cement in this situation, claimed and still claims that he only uses paste "in

* For proof of each of these positions, see an article in the Journal, Vol. 4, No. 3, p. 175, &c.

certain cases," and we are inclined to believe him, if this phrase is to be placed in contra-distinction to *un*certain cases, for surely there was no *un*certainty connected with it. We do not introduce this case, however, intending to carry the idea that this is the practice of all who use amalgams, but simply to show, that should a Society prohibit its use "except in certain cases," it would be no prohibition whatever—nay, it would be nothing less than a perfect burlesque.

We might here safely close our review of Dr. Allen's letter, as nearly all his subsequent remarks and inferences are intimately connected with, and mainly depend upon his first position, which we have shown not to be founded on facts, nor on even a reasonable hypothesis. But as his communication notices several objections to the proceedings of the Society, which have been also urged by others, and probably will be, by all who refuse to comply with the requirements of the Society, we shall give them a passing notice. We will next notice Dr. Allen's great surprise on receiving the protest of the Society. He says, "just as we were congratulating ourselves upon the restoration of harmony among the different members, lo! its ghost appeared in the form of a protest." Was this an unlooked for document? It certainly is to be presumed that Dr. A. knew what action was taken upon this subject by the Society, at the last meeting. He was present more or less during the session; and it would be unreasonable to suppose that he did not learn the result of the deliberation, after taking a deep interest in the subject. If so, he must have known that certain resolutions were passed upon this subject, and among others, one which stated definitely, that such a circular should be issued by the Society, and sent to every member for his signature. It directed "that the recording secretary be requested at the same time to transmit to the several members as aforesaid, *a printed protest or certificate, in accordance with the above named resolutions*," &c.

Now, on the supposition that Dr. A. knew what resolutions were passed at this meeting, we are constrained to conclude that he not only expected that *a* certificate would be presented, for his signature, but that he also must have known precisely what *kind* of a certificate this would be, viz. one *in accordance with* the resolutions, which were in every particular most explicit. But we are not wholly left to conjecture upon this point. In a note to Dr. A's letter, he informs us that three, viz. John Lovejoy, Geo. E. Hawes and Chas. C. Allen, communicated to the Society their intention to discontinue its use for the future, and speaks of the circumstance that these communications were not published with the transactions of the Society, as "manifestly unfair." We will now correct this oversight as far as possible, by giving these communications a place here. We shall learn, moreover, from Dr. Allen's communication on this occasion, that he was by no means ignorant of the deliberations of the Society upon this subject. His letter reads as follows:

"*Mr. President, and Members of the American Society of Dental Surgeons:*

"When first I learned that for the purpose of suppressing malpractice in dentistry, this Society had decided that *each and every member should pledge himself, by his signature, not to use the amalgam upon the teeth, in any case whatever*, I did not see how I could consistently comply with the requisition, for the following reasons:

"1st. I considered it unwise and arbitrary for this Society to pass a resolution that a certain course, which many of its members were pursuing, was malpractice, and disciplining those members, without proving that injury had been done to their patients by such practice.

"2d. From circumstances which had come to my knowledge, it appeared to me like exposing the quarrels of some members of the Society, who had to contend with rivals who were in the practice of using this proscribed article, which, if true, I considered incompatible with the high and dignified stand which I wished this Society to take.

"3d. I could not but consider it an unwarrantable attack upon the practice of many members of the Society, who were honestly using the amalgam for the benefit of their patients.

"4th. I doubted if it would produce the desired effect—that of suppressing quackery and imposture—and feared that the many educated and honest dentists, not members of the Society, who are daily using the mercury and silver, would plead to their patients, and at the bar of public opinion, which I have always thought was in favor of a limited use of the amalgam, that the American Society of Dental Surgeons was proscribing and persecuting them, because they would not become members, and submit to be dictated to by the Society.

"5th. I had myself used the article for more than eight years, never from choice, but only when I could use neither gold nor tin; in all these cases I had used it experimentally, watching the effect of the operation with great care, and I can now truly say, that I have never seen any bad effects result from its use, which I have not seen from the use of both gold and tin; but that, in many cases, it has preserved the teeth, strengthening and making them useful organs of mastication for many years. Entertaining these views, I felt exceedingly unwilling to abandon the use of the amalgam; but as many of the members of this Society, who have used this article more than I have, and for whose opinion I have the highest regard, have decided to use it no more, and as I do not wish to impose the slightest obstacle to the success, union and harmony of the Society, I cheerfully consent to do the same, hoping that all the members will feel the importance of making mutual concession, if they would be a happy, useful and prosperous Society.

"Very respectfully,

CHAS. C. ALLEN."

The following is the communication referred to, from John Lovejoy:

"*Brothers of the American Society of Dental Surgeons:*

I was present this morning, when the letter of our worthy brother Dr. Allen was read. It contains exactly my views in relation to cement and its use. I, with him, hereby cheerfully say, that I will henceforth use it not—not because I consider its use, under all circumstances, malpractice, but because I consider it my duty to make concessions to the honest views and desires of a majority of my brothers of the Society, and to give greater efficiency to its acts. Be assured, my brothers, that I

have none but the kindest feelings towards every member. My heart and hand shall go with you.

[Signed,] JOHN LOVEJOY."

"NEW YORK, *8th Aug.*, 1845."*

Next in order, Dr. Allen calls in question the constitutionality of this act of the Society, and in doing this, he certainly starts with a very fair proposition—one which he may, not only with a great deal of assurance, but with no less of reason, presume that no one will deny. To all his deductions, however, we are not quite so well prepared to yield a ready assent. He says, "I presume it will not be denied by any member of our Society, that the first law of our association, under which it was formed, and without which it could not well exist, is the *constitution*, and that all the provisions contained therein are inviolate, and cannot be changed, except in the manner prescribed therein." "If this proposition be true," he adds, "then it follows that no resolution passed by a majority, I care not how large, is of any binding force upon the members, if the *principle* contained in the said resolution conflicts with the *principles* laid down in the constitution." This we fully grant, and shall take the liberty to add another deduction, which *we presume* will not be denied by any member of the Society, viz. any resolution passed by the Society, *not conflicting* with the spirit and "true intent" of the constitution, *is* constitutional, and *is of binding force* upon every member.

Now the formation and existence of any association necessarily implies an object or a *main end* to be gained. The constitution of such society, including its preamble, is merely to set forth such object and embody such general rules and precepts as shall govern such society in securing such object. An association may, it is true, have secondary purposes, but these must be collateral and tributary to the main end—certainly they must not conflict with it. We may illustrate our proposition by familiar cases. For example, a religious association might properly deny membership to one who was intemperate, for the very obvious reason that intemperance conflicts with the object

* Dr. Hawes' letter is not in our possession.

of the association; but, on the other hand, a temperance association could not, with any show of justice, return the compliment, for the simple reason that, although there can be no higher object than the promotion of true religion, yet it is not *essential* to temperance. Nor could either society refuse membership to a man, in other respects eligible, on account of his *political* faith. Nor is it necessary that these specific cases be mentioned in the constitution of the society, or creed of the church, 'tis enough that the transaction harmonizes with their spirit and intent. Let us now inquire, what is the main object or end of the American Society of Dental Surgeons, and whether their action upon the subject of amalgams is at variance with their prerogatives, according to the true intent of the constitution. If it is, then have the Society "transcended their powers;" but if not, their measures are constitutional.

The object which this association had in view at the time of its formation, and which of course remains the same, is distinctly set forth in the preamble to the constitution. This, after enumerating several important aims, sums up the whole, both as to the *object* and the manner of attaining that object, in the following brief but explicit language, viz. the object is, "in fine, *to give character and respectability to the profession, by establishing a line of distinction between the truly meritorious and skilful, and such as riot in the ill-gotten fruit of unblushing impudence and empiricism.*" We have only now to inquire whether fellowshiping those who persist in a practice which the Society have declared, time and again, to be *unqualifiedly mal*-practice, would be in accordance with the object as above set forth; or rather, whether it would not be in direct violation of that object.

But we are very gravely told, that the Society have no *right* to condemn *any* practice, "*a priori,*" as malpractice; that it "would establish a precedent fraught with innumerable evils." Our correspondent greatly fears that if the Society condemn the use of mineral paste, "*a priori,*" and expel members for this practice, that "another batch will probably be expelled, whenever the Society see fit to correct the abuse of arsenic"—"the turnkey," &c. Now to such logic we think there is a two-fold objection. That a society, whose *main end* is to correct abuses connected with the dental profession, should be denied their prerogative to correct one abuse, because it would be establishing such a precedent as would require them to attack a second, and a third, is a doctrine new and peculiar, to say the least. For our own part, when the proof is as clear that "arsenic," "the turnkey," "kreosote," or any other article, is never called for, is never efficient in accomplishing the end for which it is used, is uniformly bad in its tendency, and liable, moreover, to produce dangerous results in the hands of *any* practitioner, as it is relative to these objectionable features of amalgams, then we shall certainly give our vote, not only to condemn them, but to expel such members of the Society as persist in their employment.

But we are still of the opinion, that the Society *have* a *right* to condemn a *practice* on good evidence, notwithstanding "our friend, the doctor," thinks the constitution expressly "secures to each member the right to advocate any principle or *sentiment* connected with dentistry." The following quotation from the preamble, he makes to justify this conclusion, viz. "that one of the objects of the American Society of Dental Surgeons is *to advance the science* (of dentistry) by *free* communication and interchange of sentiments;" &c. Here the preamble very clearly presents the main end, *the advancement of the science of dentistry*, and it as clearly sets forth the *object* of this "*free communication*," which is to secure the attainment of this main end. Now we inquire whether it is probable that the framers of this instrument anticipated the doctrine Dr. Allen contends for, as coming appropriately under the provision which he quotes. Did any individual ever *advance the science of dentistry*, by contending that mineral paste might, under any circumstances be used for filling carious teeth? Or has any one "advanced the science," by the *use* of this "untempered mortar?" If this is the way to advance the science, or it can be shown that a practice requiring less skill to command it fully than that possessed by a common mason, and bad in its every tendency, ever added any "character or respectability to the profession," then we will cheerfully concede the ground that the *constitution does* secure to all the privilege not only to *advocate*, but to *use* mercurial paste, to any extent that any member may please; but till then we shall subscribe to no such doctrine.

Most truly would it be wrong and arbitrary to suppress light upon *any* subject, before its real merits and demerits were fully exhibited. The constitution justifies no such doctrine, nor have the Society attempted to enforce any such sentiment. No man can claim that this subject has not been fully open for discussion; indeed, it has occupied already more pages of the Journal than any other subject. The same is true of it, as connected with our annual meetings. But a time arrived when its merits were deemed no longer worthy of discussion. It was decided that the time and attention of the Society devoted to this subject was wasted, after the practice in question was, on satisfactory evidence, *condemned.* The only remaining question, so far as the Society was concerned, was how to dispose of it, or rather how to free their ranks from its contaminating influence. This they decided to do by issuing the circular alluded to. Now it is a curious fact connected with the history of this matter, that notwithstanding this practice was attacked on all hands, and its deformities faithfully shown up, year after year, yet not an advocate appeared to defend it, till after the passage of such a law as necessarily compelled its members either to abandon it, or leave the Society. And it is more curious still, that notwithstanding every member who did *actually* use this article for dental purposes, by the Society stood charged with malpractice, year after year, yet not a single member, to our knowledge, made the least resistance to this *now* "arbitrary" decision and charge, till matters were so arranged that every member was compelled to take a stand. But no sooner did the Society decide that the house should be divided, and adopt measures which required each member either to abandon mineral paste or relinquish his membership, than the hue and cry was raised of persecution, of its being an unconstitutional condemnation. But it seems to us, that such objections, at this late day, based on the pretence that it is attempting to enforce a "gag-law," by denying the right of that "free communication" which is claimed to be gauranteed by the constitution, is the height of absurdity. It would be just as reasonable for a member of the legislature to complain that the passage of any given law was *unconstitutional,* because they did not leave it open for discussion, after it had became a law. It is an old saying, that "there is a time for all things"—a time to discuss a law, a time to enact it, and a time to execute it, and we consider the resolutions passed by the American Society, during its last session, as in their nature and tendency wholly executory. Now it seems to us, that if those whose right it was, have failed both during the discussion and during the action upon it, to dissent from any law, that it is not a time to complain of it after it has become a law, and is being executed, especially on the ground that it should be still open for discussion. This would indeed, as applied to the question raised by the advocates of mineral paste, be an accommodating feature, as it would leave them free to use it while a discussion would be perpetually kept open. The unpleasant alternative of either abandoning the practice or membership would never be enforced. But this principle, applied to our civil laws, would, as all must see, be equivalent to abolishing all law. Deny the right to execute laws, and you may, with equal safety, dismiss our legislators, and make a bonfire of our statute books. But to return to our examination of facts and circumstances connected with the transactions of the Society.

Is it in any sense fair to presume that it is construing the constitution according to its "true intent," by giving it a construction which makes it sanction, nay, recommend, a practice or its advocacy, which the Society, including those who framed the constitution, have, time and again, declared to be malpractice? Have the American Society of Dental Surgeons been so weak, or so far destitute of sagacity, as to adopt a constitution *necessarily* conflicting with the attainment of one of its chief objects? And we here repeat what Dr. A. objects to in our former communication, that the decision of the Society, in respect to this practice, viz. that it is malpractice, "every member was as much bound to adopt as any precept either in the constitution or by-laws." And why? because it involved a principle necessary to be sustained, in order that the Society might secure one of their most prominent objects—the suppression of quackery. Is not every member bound to assist in carrying out the *objects* of the Society? To subvert this position,

Dr. A. reasons thus: "Now suppose a resolution were to be passed by the Society, that in its opinion hereafter the Society had better meet every year at Baltimore, instead of New York, and that one of you gentlemen, being a northern man, should vote in the negative, would you be as much bound to adopt this *opinion*, as any precept in either the constitution or by-laws?" We answer, no, by no means. There is an entire want of parallelism in the two cases. The one embraces a doctrine necessarily connected with, and involving the security of the main ends of the Society; the other has nothing whatever to do with them. When Dr. A. will show us that for the Society to meet at *any* given place is requisite, in order to secure the plainly avowed objects of the Society, then will we assent to his logic, and shall, moreover, contend that this would create a bond upon each member. Dr. A's next position, relative to the rights of a member of a church, in respect to his views upon the use of wine at the communion table, may be disposed of in the same way. There is no parallel between expelling a member of the American Society of Dental Surgeons, for a practice *never* in good repute, and excommunicating a member of a church, for a belief based on sacred writ. Indeed, the question, so far as our acquaintance extends, has never been, whether wine, in the ancient meaning of the term, should be proscribed, but simply whether our *manufactured* article, called wine, was a fit *substitute* for the juice of the grape—the article which was undoubtedly originally used and sanctioned by scripture.

But let us examine this doctrine of condemning a practice "*a priori*," in another light. Would a legislature have a right to condemn it, and to pass a law, making the use of this article for dental purposes a penal offence? We think no one will deny such a right to the legislature. We will suppose, then, such a law to be in effect, and an individual was accused of the offence, we ask, what would be the inquiry instituted? would it be how much damage had been done, or rather would it not be simply whether the individual was guilty or innocent? The former question could only come up as a measure of the punishment, but punished he must be to the minimum limit, without any reference to such an investigation. Now there are some practices connected with the medical profession, which have been thus acted upon by the legislature. The practice of producing abortions, when not necessary to save the life of the mother, has been deemed by our legislature a penal offence. Every medical society views this practice in the same light, and of course expels its members for such an offence. But is legislative action necessary to give a medical society power to condemn this practice as bad? Certainly not. But rather, has legislative action been based on testimony coming through this avenue—the concurrent and joint testimony of the profession. But even laying aside every foregoing consideration and calling it an "unprecedented act," we claim that the constitution gives the fullest liberty to condemn the practice, and expel members under that decision. Now the Society contemplates such a thing as malpractice, and fully provides for expelling members therefor. A practice may be such, either by using bad materials for any given purpose, or by using good materials badly. Now suppose any member should habitually plug lateral cavities in front teeth with *gold*, and perform the operation by pressing it in firmly between the teeth, and rely upon each tooth to hold the gold in the cavity of the other, would not the Society have the power to condemn such a practice "*a priori?*" Or would they have first to *try* each member guilty of such practice, and find how much damage he had done by his operations? Suppose again, that a member should be found, who should be in the practice of *transplanting teeth*, would Dr. Allen contend that this practice could not be condemned "a priori?" By admiting everybody into our ranks, and retaining all, we might perhaps produce *union*, but not "harmony," at least we think not that kind of harmony which our friend considers one of the leading objects of the Society. If the *Society* have not the power to decide what is, or what is not malpractice, then there can be no umpire. If she has not the power to decide upon the merits of a practice, and to expel members under such general decision, she certainly has not the power of existence; for the supposition that she must be compelled to travel over the whole globe, whither her members are scattered, and to hunt up individual cases, and "*prove* that wrong has been done," after it is fully established in respect to the *whole* practice as such, that it is uniformly bad, would be "nonsensical and absurd." But the constitution not only declares that members may be expelled for malpractice, but also that they may be expelled "for *other sufficient cause*." Now if Dr. A. is disposed to scold us for denouncing the use of mineral paste as *malpractice*, we will accommodate his taste, by putting this practice under the head of "*other sufficient cause*" for expelling a member. Now who are to decide what constitutes such a cause? The Society have unanimously decided what constituted, in their minds, *a* sufficient cause, and have, as we think, acted accordingly. Now the truth is, that the framers of the constitution, foreseeing the absolute necessity that the Society should have the most ample powers vested in them, to purify their ranks, after making what might be deemed full provision for this, superadded this indefinite clause, that there might be none who could evade its decisions, confined under definite heads; and we contend we are fully justified in saying this covers all the ground we claim, were we to abandon every other.

There can, then, exist no reasonable doubt that the Society have a right to condemn a practice, and of course to condemn the practice in question; nor is there any more doubt in respect to the right to expel members for persisting in a course which they have condemned.

But Dr. Allen has found another "lion in the way," or as we may better say, another "straw," which he holds on to with a lion's grasp. 'Tis an old saying, and as true as old, that "necessity is the mother of invention," and it is only on this principle that we can account for the extreme ingeniousness of the inventions of our correspondent, to make it appear that the Society have acted unwisely and unconstitutionally, and hence that their action is not of binding force. He has now found a flaw in the *manner* in which this business has been done. He says these persons "were either expelled at the last regular meeting, or they were not;" "if they were not expelled," he adds, "then they cannot be till the next regular meeting." But has not "our friend, the doctor," with all his erudition upon legal criticism, heard of such a thing as conditional expulsion, to take place in future? Suppose a society of the Sons of Temperance find that one of their members has broken his pledge, would not this association have an undoubted right to declare him expelled, unless he renew his pledge, and make certain acknowledgments by a certain time? Would it be necessary *actually* to expel him, and then go through the process of again receiving him, should he comply with the requisition, in order to have the thing done constitutionally?

How common is it that a member of a church is thus suspended—to be, on some future day, either restored to full fellowship by compliance, or cut off by non-compliance with a similar decree.

A case involving a similar principle in respect to conditional punishment, occurred not long since in our own state, where the consequences were much more momentous. A man was charged with crime, tried, convicted, and sent to Auburn state's prison. Governor Seward pardoned him, *on condition* that he would leave the state, and never return; but that, in case he did return, his original sentence was to be in full force. Now this man obtained his liberty under this conditional act, and left the state; but at length, feeling that his liberty was curtailed, ventured to return. The result was that he was retaken, and by virtue of his original sentence re-imprisoned. Now Dr. Allen's reasoning, applied to this case, would evidently be this: This man was either pardoned, or he was not; if he *was* pardoned, then the simple act of his refusing to leave the state could not make him again a criminal under sentence, till he had a new trial and a new sentence; if he was not pardoned by this conditional decree, then his leaving the state, (allowing he might effect his escape,) would not remove the sentence of the law. But notwithstanding the sageness of such a doctrine, this man was released by virtue of this conditional pardon, on his compliance with the condition, and was again imprisoned on his forfeiting this condition, and this too, without any new trial for the crime.

But why need we travel abroad for precedents and authority, when we have both in abundance at home. The constitution of our own Society gives explicit and full power to expel conditionally, and by virtue of this power the Society have expelled several of its members.

The treasurer has at all times had the power to "strike from the roll" those members who, "on being notified, neglect to pay their annual dues for three meetings" of the Society.* At the last annual meeting several were reported as expelled, in consequence of this neglect to pay dues, and they now stand recorded as expelled.

But we will not waste words upon this part of our subject. Dr. Allen admits that the Society had a right to expel these members at the last meeting, it being a "regular meeting," and for him to complain of the very *lenity* shown by the Society, that all might have ample notice and time to decide as to the course they preferred to take in this matter, and moreover to make it a ground of unconstitutionality, is a feature we are at a loss to know how to construe, unless we take him at his word, and adopt the belief that he is *still* "exceedingly unwilling to abandon the use of amalgams," while he is at the same time as "unwilling" to be deprived of the distinction which membership gives him.

We would not be uncharitable, yet we can truly say, that in our circle of acquaintance we have never seen an instance where an individual considered it his privilege even to *advocate* the use of amalgams, where his reputation was not to a considerable extent identified with this practice, and where his reputation would be implicated were the practice to be condemned.

### ARTICLE II.

*Observations on the Evils Resulting from the Premature Application of Artificial Teeth on Plate, with Clasps, Obturators, Palates, &c.* By JOHN HARRIS, M. D., D. D. S., late of Georgetown, Ky.

THE resources of art, in supplying defects of the body occasioned by the ravages of disease, or physical or accidental causes,

* Art. 4, sec. 3, Revised Constitution.

